# Influence of varying oral pH conditions on the material stability of thermoplastic orthodontic aligners: an in vitro study

**DOI:** 10.1186/s12903-026-08272-z

**Published:** 2026-05-15

**Authors:** Dhruv Ahuja, Bhawna Negi, Anjana Goyal, Puneet Batra, Ashith MV, Kumar Rakshak Anand, Khushi Gupta, Bhoomi Pitanjali Singh, Areeba Parvez

**Affiliations:** 1 Department of Orthodontics and Dentofacial Orthopedics, Manav Rachna Dental College, Manav Rachna International Institute of Research and Studies (MRIIRS), Faridabad, Haryana India; 2 Manav Rachna Dental College, Manav Rachna International Institute of Research and Studies (MRIIRS), Faridabad, Haryana India; 3 Department of Biochemistry, Manav Rachna Dental College, Manav Rachna International Institute of Research and Studies (MRIIRS), Faridabad, Haryana India; 4 Department of Orthodontics and Dentofacial Orthopedics, Manipal College of Dental Sciences Mangalore, Manipal Academy of Higher Education, Manipal, Karnataka 576014 India; 5 Department of Oral and Maxillofacial Surgery, Manav Rachna Dental College, Manav Rachna International Institute of Research and Studies (MRIIRS), Faridabad, Haryana India

**Keywords:** Clear aligners, Thermoplastic polymers, Colour stability, Leaching, Chemical exposure, In vitro study

## Abstract

**Background:**

Clear thermoplastic orthodontic aligners are widely used due to their aesthetic appeal and patient comfort; however, exposure to chemically diverse intraoral environments may compromise their physical, aesthetic, and chemical stability. This study aimed to evaluate the effect of acidic, alkaline, and neutral chemical environments on weight variation, pH interaction, colour stability, and leaching behaviour of thermoplastic orthodontic aligner materials.

**Methods:**

Thirty maxillary thermoplastic orthodontic aligners representing three commercial materials (Erkodur, Duran, and Zendura) were immersed in acidic (lime juice), alkaline (ENO^®^ solution), or neutral (artificial saliva) media for 14 days at 37 °C. Assessments were performed at Day 0, Day 7, and Day 14. Weight changes, pH variation, colour stability (ΔE, CIE L*a*b* using ImageJ and VITA Easyshade), and leaching behaviour (UV-Visible spectrophotometry) were analysed using repeated-measures and one-way ANOVA.

**Results:**

Aligners in acidic media exhibited the greatest weight reduction (3.410 ± 0.015 g to 3.358 ± 0.025 g; *p* < 0.001), followed by alkaline media. Colour change was highest in acidic conditions (ΔE = 3.08 ± 0.15), moderate in alkaline (1.92 ± 0.12), and minimal in neutral media (0.77 ± 0.08). Leaching absorbance was significantly higher in acidic media at Day 14 (0.083 ± 0.004 AU; *p* < 0.001).

**Conclusion:**

Oral chemical exposures significantly compromise the physicochemical and aesthetic properties of thermoplastic aligners, highlighting the need for careful material selection, patient guidance, and further in vivo studies to confirm clinical impact.

## Introduction

Clear thermoplastic orthodontic aligners and retainers have become a cornerstone of modern orthodontic practice due to their superior aesthetics, comfort, and patient acceptance compared with conventional fixed appliances [1]. These appliances are typically manufactured from amorphous thermoplastic polymers such as polyethylene terephthalate glycol (PETG), thermoplastic polyurethane (TPU), and related copolymers, which provide high translucency, flexibility, and mechanical adaptability [2].

Once placed in the oral cavity, aligners are exposed to a dynamic environment involving mechanical forces, temperature fluctuations, salivary moisture, enzymatic activity, and variable pH, all of which can compromise material stability and function [3, 4]. Intraoral aging has been shown to cause changes in water absorption, dimensional integrity, and optical properties, including loss of clarity and surface discoloration, potentially affecting appliance performance over time [5].

Colour stability and dimensional integrity are critical because they influence both aesthetic outcomes and appliance functionality. Discoloration not only reduces visual appeal and patient satisfaction but may also indicate underlying material degradation affecting transparency and aligner lifespan [6]. Weight variation, often due to water uptake or interaction with oral fluids, may reflect changes in polymer morphology that can alter fit and force delivery [7]. Additionally, thermoplastic polymers may leach low-molecular-weight components, including additives or residual monomers, raising concerns about chemical stability and biocompatibility [8].

Although certain thermoplastic polymers may release trace amounts of residual monomers or additives under specific conditions, the extent and clinical relevance of such release from orthodontic aligners remain an area of ongoing investigation, particularly with respect to cumulative exposure during long-term treatment [4]. Despite these insights, existing literature has primarily focused on isolated outcomes, such as colour change or water absorption, without concurrently evaluating multiple physicochemical parameters, including weight variation, pH interaction, aesthetic stability, and leaching, under controlled conditions [3, 7]. Furthermore, in vivo data suggest that intraoral aging adversely affects aligner properties, yet systematic comparative studies across diverse chemical exposures remain limited [9]. Compromised structural or aesthetic stability may affect patient satisfaction, appliance longevity, and treatment outcomes. For instance, water absorption and dimensional changes can alter the force delivery of aligners, potentially reducing treatment predictability [10].

Similarly, visible discoloration or material degradation may diminish patient compliance and perceived treatment value [5]. Addressing these gaps is essential for optimizing material selection, aligner design, and evidence-based guidance for patient care during aligner therapy. Accordingly, the aim of the present in-vitro experimental study was to evaluate the effect of chemical exposure on key physical and chemical properties of thermoplastic orthodontic aligner materials. The null hypothesis was that these exposures would not result in significant changes in weight, immersion media pH, colour stability, or leaching behaviour over time.

## Materials and methods

This in-vitro experimental prospective study was conducted to evaluate the effect of different chemical environments on different orthodontic aligner materials. As the investigation did not involve human participants, animal subjects, or identifiable biological samples, institutional ethical approval was not required. The study assessed changes in weight, pH of the immersion media, colour stability, and leaching behaviour of thermoplastic orthodontic aligners at Day 0, Day 7, and Day 14 under controlled laboratory conditions.

### Sample

The sample size was calculated using G*Power software (version 3.1.9.7) for comparison of three independent groups with repeated measurements over time. An effect size of 0.40, power of 80%, and alpha error rate of 5% were assumed based on previously published in-vitro aligner studies. The minimum required sample size was calculated to be 30 aligner samples per group, with 10 samples allocated to each group. All measurements were performed in triplicate to enhance reliability.

Thirty maxillary thermoplastic orthodontic aligners representing three commonly used commercial materials (Erkodur(Erkodent Erich Kopp GmbH, Germany), Duran (Scheu-Dental GmbH, Germany), and Zendura (Bay Materials, LLC, California)), each obtained from a single production batch per brand, were included to minimize intra-brand variability and allow controlled assessment of chemical exposure effects across different aligner materials. The aligners were randomly assigned to three experimental groups based on the immersion medium: acidic medium (lime juice), alkaline medium (ENO® fruit salt solution), and neutral medium (artificial saliva). Each aligner remained in its assigned medium throughout the 14-day study period and was evaluated longitudinally at Day 0, Day 7, and Day 14. Data from the three aligner materials were pooled for analysis as the primary objective of the study was to evaluate the effect of chemical environment rather than inter-material differences. Pooling was performed after confirming that no significant baseline differences were observed among the materials.

### Preparation of immersion media

**Fig. 1:**
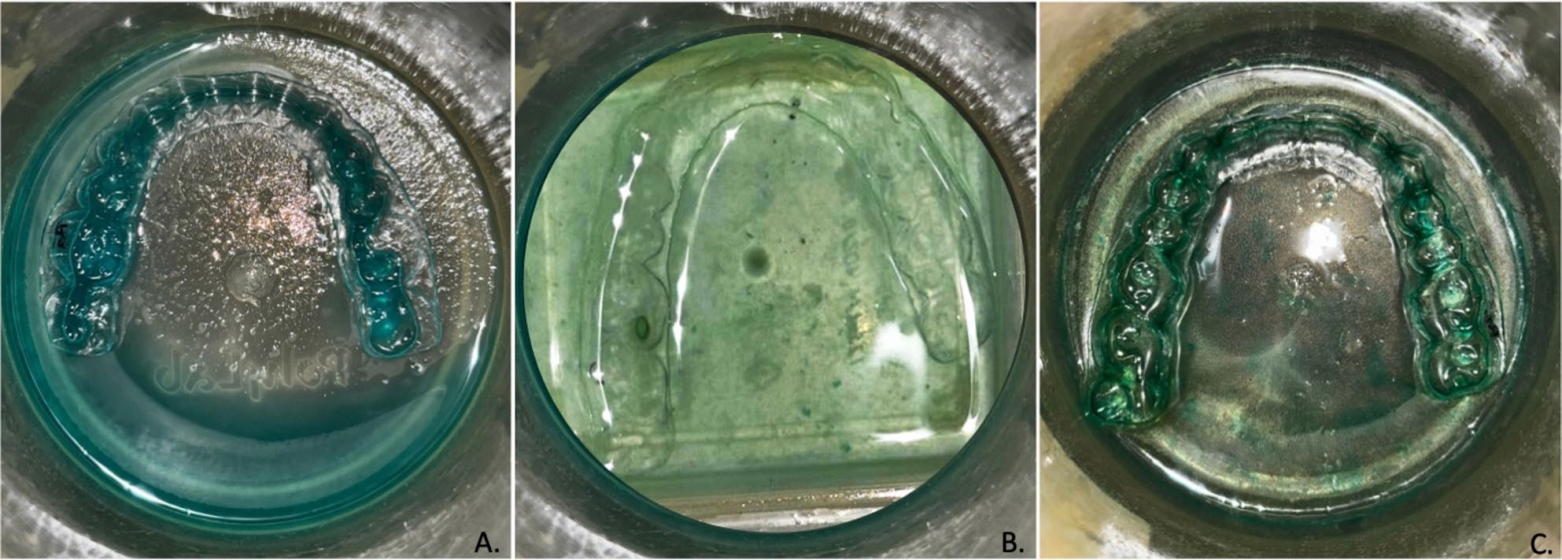
Immersion media used for aligner exposure: **A** acidic (lime juice), **B** neutral (artificial saliva), and (**C**) alkaline (ENO^®^ solution)

The acidic medium consisted of freshly prepared lime juice, filtered to remove pulp and diluted with distilled water in a 1:1 ratio to standardise acidity (pH recorded at baseline) [11]. The alkaline medium was prepared by dissolving ENO^®^ fruit salt (GlaxoSmithKline, India) in distilled water at a concentration of 5 g per 200 mL, following manufacturer instructions [12]. The neutral medium comprised artificial saliva prepared using a standard formulation containing sodium chloride, potassium chloride, calcium chloride, sodium dihydrogen phosphate, and urea, adjusted to neutral pH to simulate oral conditions [13, 14]. (Fig. 1)

### Immersion protocol

Each aligner was completely immersed in 50 mL of the respective solution in sterile borosilicate glass containers. Containers were sealed and stored in a laboratory incubator at 37 ± 1 °C to simulate intraoral temperature. Aligners were continuously immersed for 14 days without agitation. At each evaluation point, specimens were removed, rinsed with distilled water, and air-dried prior to testing [15]. 

### Weight measurement

Aligner weight was measured at each time point using a digital analytical balance (Shimadzu AUX220, Shimadzu Corporation, Japan; accuracy ± 0.001 g). Prior to weighing, specimens were blotted dry with lint-free tissue and allowed to equilibrate at room temperature for 5 min to eliminate surface moisture. Each measurement was recorded in triplicate, and the mean value was used for analysis [16]. 

### pH measurement of immersion media

The pH of each immersion medium was measured at Day 0, Day 7, and Day 14 using a calibrated digital pH meter (Eutech Instruments pH 700, Thermo Fisher Scientific, USA). Calibration was performed using standard buffer solutions at pH 4.0 and pH 7.0 prior to each measurement session. Changes in pH were recorded as indicators of chemical interaction between aligner materials and the immersion media [17]. 

### Colour stability assessment

Colour stability was evaluated using a standardized visual and photographic assessment protocol. Baseline colour documentation was performed prior to immersion, followed by evaluations at Day 7 and Day 14. All aligners were photographed under standardized lighting conditions against a neutral background using a fixed digital camera setup (DSLR camera, Canon EOS 80D; Canon Inc., Tokyo, Japan) with identical exposure, aperture, and white balance settings to minimize variability. The camera-to-sample distance, angle of capture, and lighting geometry were kept constant using a fixed mounting system. All images were captured in the same session using identical camera settings (ISO, aperture, shutter speed, and white balance), and no post-processing adjustments were applied prior to analysis. Colour measurements were performed by a single calibrated examiner to minimize operator-related variability. The captured images were analysed using VITA Easyshade and digital image analysis software (ImageJ, version 1.53; National Institutes of Health, Bethesda, MD, USA) to quantify changes in translucency and colour parameters based on the CIE L*a*b* colour system. Colour differences (ΔE) between time points were calculated using the standard formula: ΔE=(L2 − L1)^2^+(a2 − a1)^2^+(b2 − b1)^2^ [18, 19].

### Leaching evaluation

Leaching of substances from aligner materials was assessed using a solution-based analytical approach adapted from previously published orthodontic aligner studies. After each immersion period, the storage solutions were collected and analysed using UV–Visible spectrophotometry (UV-1800, Shimadzu Corporation, Japan) over a wavelength range of 200–400 nm to detect the presence of leached organic compounds. Absorbance values were recorded and compared across time points and groups. Increased absorbance was interpreted as indicative of the presence of soluble substances released from the aligner material. However, UV-Visible spectrophotometry provides only a preliminary indication of leaching and does not allow identification or quantification of specific compounds. Therefore, the observed absorbance values likely represent a mixture of low-molecular-weight components released from the polymer matrix. This approach has been previously employed for preliminary assessment of chemical stability in thermoplastic orthodontic appliances [20]. 

### Statistical analysis

All data were entered into IBM SPSS Statistics version 26.0 (IBM Corp., Armonk, NY, USA) for analysis. Normality of the data was assessed using the Shapiro–Wilk test. Repeated measurements over time (Day 0, Day 7, Day 14) were analysed using repeated-measures analysis of variance (ANOVA) to compare changes within each group, and one-way ANOVA with Tukey’s post hoc test was used to compare differences between groups at each time point. Data from the three aligner materials were pooled for analysis, as the primary objective was to evaluate the effect of chemical exposure rather than inter-brand differences. For colour stability and leaching, ΔE values and absorbance readings were treated as continuous variables. Weight and pH changes were similarly analysed. Triplicate measurements were averaged for each sample to ensure reliability.

## Results

### Weight changes

Baseline weights were similar across groups. Over 14 days, aligners in acidic medium showed the greatest weight loss (3.410 ± 0.015 → 3.358 ± 0.025 g), followed by alkaline (3.411 ± 0.016 → 3.386 ± 0.022 g), while neutral medium remained nearly unchanged (3.409 ± 0.015 → 3.400 ± 0.017 g). Repeated-measures ANOVA revealed significant within-group effects for acidic (F = 56.42, df = 2,18, *p* < 0.001) and alkaline media (F = 21.36, df = 2,18, *p* = 0.002), but not neutral (F = 1.25, df = 2,18, *p* = 0.32). Between-group differences at Day 14 were significant (F = 34.21, df = 2,27, *p* < 0.001; Table 1).

**Table 1:** Changes in Aligner Weight Over Time (g)

Medium	Day 0 (g)	Day 7 (g)	Day 14 (g)	Within-Group (Time Effect) (df), *p*, η²	Between-Group F (df), *p*, η² (Day 14)
Acidic	3.410 ± 0.015	3.396 ± 0.021	3.358 ± 0.025	56.42 (2,18), < 0.001*, 0.966	34.21 (2,27), < 0.001*, 0.919
Alkaline	3.411 ± 0.016	3.403 ± 0.020	3.386 ± 0.022	21.36 (2,18), 0.002*, 0.914	
Neutral	3.409 ± 0.015	3.402 ± 0.016	3.400 ± 0.017	1.25 (2,18), 0.32, 0.385	

F = test statistic, df = degrees of freedom, η² = effect size

*p* < 0.05 considered statistically significant

### pH changes

Acidic medium increased slightly from 3.8 ± 0.1 to 4.1 ± 0.1, alkaline decreased from 8.2 ± 0.2 to 7.9 ± 0.1, and neutral remained stable (7.0 ± 0.1). Within-group ANOVA showed significant changes for acidic (F = 12.48, *p* = 0.004) and alkaline media (F = 7.36, *p* = 0.018), but not neutral (F = 0.02, *p* = 0.98). Between-group differences at Day 14 were significant (F = 15.67, *p* = 0.002; Table 2).

**Table 2:** Changes in Immersion Media pH Over Time

Medium	Day 0 (pH)	Day 7 (pH)	Day 14 (pH)	Within-Group (Time Effect) (df), *p*, η²	Between-Group F (df), *p*, η² (Day 14)
Acidic	3.8 ± 0.1	3.9 ± 0.1	4.1 ± 0.1	12.48 (2,18), 0.004*, 0.862	15.67 (2,27), 0.002*, 0.892
Alkaline	8.2 ± 0.2	8.1 ± 0.1	7.9 ± 0.1	7.36 (2,18), 0.018*, 0.787	
Neutral	7.0 ± 0.1	7.0 ± 0.1	7.0 ± 0.1	0.02 (2,18), 0.98, 0.010	

F = test statistic, df = degrees of freedom, η² = effect size

*p* < 0.05 considered statistically significant

### Colour stability (ΔE)

Acidic medium showed the largest colour change (ΔE Day 14: 3.08 ± 0.15), followed by alkaline (1.92 ± 0.12), and minimal change in neutral (0.77 ± 0.08). Repeated-measures ANOVA confirmed significant within-group effects for acidic (F = 48.37, *p* < 0.001) and alkaline (F = 22.54, *p* = 0.001), but not neutral (F = 2.16, *p* = 0.18). Between-group differences at Day 14 were significant (F = 37.65, *p* < 0.001; Table 3).

**Table 3:** Colour Change (ΔE) Over Time

Medium	Day 0 (ΔE)	Day 7 (ΔE)	Day 14 (ΔE)	Within-Group (Time Effect) (df), *p*, η²	Between-Group F (df), *p*, η² (Day 14)
Acidic	0	1.97 ± 0.13	3.08 ± 0.15	48.37 (2,18), < 0.001*, 0.960	37.65 (2,27), < 0.001*, 0.926
Alkaline	0	1.44 ± 0.11	1.92 ± 0.12	22.54 (2,18), 0.001*, 0.918	
Neutral	0	0.69 ± 0.09	0.77 ± 0.08	2.16 (2,18), 0.18, 0.519	

F = test statistic, df = degrees of freedom, η² = effect size

ΔE is based on the *CIE Lab colour system*

*p* < 0.05 considered statistically significant

### Leaching (UV–Visible Absorbance)

Absorbance increased most in acidic medium (Day 14: 0.083 ± 0.004 AU), moderately in alkaline (0.054 ± 0.003 AU), and minimally in neutral (0.022 ± 0.002 AU). Within-group ANOVA indicated significant effects for all media except neutral (acidic: F = 79.28, *p* < 0.001; alkaline: F = 31.62, *p* < 0.001; neutral: F = 5.14, *p* = 0.049). Between-group differences at Day 14 were significant (F = 42.15, *p* < 0.001; Table 4).

**Table 4:** UV–Visible Absorbance (AU) Over Time

Medium	Day 0 (AU)	Day 7 (AU)	Day 14 (AU)	Within-Group (Time Effect) (df), *p*, η²	Between-Group F (df), *p*, η² (Day 14)
Acidic	0.005 ± 0.001	0.062 ± 0.003	0.083 ± 0.004	79.28 (2,18), < 0.001*, 0.975	42.15 (2,27), < 0.001*, 0.934
Alkaline	0.004 ± 0.001	0.043 ± 0.002	0.054 ± 0.003	31.62 (2,18), < 0.001*, 0.941	
Neutral	0.003 ± 0.001	0.018 ± 0.002	0.022 ± 0.002	5.14 (2,18), 0.049*, 0.720	

F = test statistic, df = degrees of freedom, η² = effect size

UV–Visible absorbance measured at 200–400 nm wavelength range

*p* < 0.05 considered statistically significant

## Discussion

This in-vitro study demonstrates that exposure to different chemical environments significantly affects the physicochemical properties of thermoplastic aligner materials, including weight, immersion media pH, colour stability, and leaching behaviour. Aligners immersed in acidic media exhibited the greatest weight loss and colour change over 14 days, followed by alkaline media, while neutral conditions showed minimal alterations. UV–Visible absorbance (leaching) was also significantly higher in acidic environments, indicating greater release of low-molecular-weight components.

These findings align with previous in-vitro and in-vivo research showing that clear orthodontic aligners undergo measurable changes when exposed to oral conditions or staining agents. Liu et al. reported that various solutions, including beverages and cleaning agents, induce significant colour changes in thermoplastic aligners, highlighting material susceptibility to aesthetic degradation under prolonged exposure [21]. Similarly, studies on clinical aging of clear aligners have documented optical and translucency changes as well as increased water absorption and dimensional variation, which correlate with alterations in mechanical and physical properties over time [22]. 

Colour stability has been a focal point in recent aligner research because aesthetic integrity influences patient satisfaction and compliance. In vitro studies exposing aligners to indigenous food products and beverages demonstrated significant discolouration, particularly with compounds like coffee and turmeric, confirming that everyday dietary habits can compromise aesthetic qualities [19]. These reports support our observation of greater ΔE in acidic conditions, underscoring the clinical relevance of advising patients on exposure to potentially staining solutions.

In interpreting these findings, it is important to consider established thresholds for clinically perceptible colour change. Previous studies have suggested that ΔE values between approximately 1.2 and 2.7 represent the perceptibility threshold for the human eye, while values exceeding approximately 3.3 may be considered clinically unacceptable for dental materials [23]. In the present study, the ΔE value observed in acidic conditions approached this acceptability threshold, whereas colour changes in alkaline and neutral media remained within or below perceptible ranges. These findings suggest that prolonged exposure to acidic environments may have greater potential to compromise the aesthetic appearance of clear aligners during clinical use.

The observed changes in immersion media pH reflect chemical interactions between the polymer matrix and the surrounding environment. While neutral media remained stable, slight increases in acidic conditions and decreases in alkaline conditions suggest reactive processes at the polymer–medium interface. This is consistent with polymer chemistry studies showing that thermoplastic materials can undergo hydrolytic and sorption changes depending on medium pH and composition, ultimately influencing both aesthetic and structural stability [4, 24]. 

These changes may be explained by the susceptibility of thermoplastic polymers such as PETG and polyurethane to hydrolytic degradation under extreme pH conditions. Acidic environments may promote cleavage of ester bonds within the polymer backbone, leading to gradual polymer chain degradation and release of low-molecular-weight fragments. Similarly, alkaline conditions may facilitate polymer swelling and water sorption, altering the structural integrity of the material. Such physicochemical interactions can contribute to weight variation, optical changes, and increased release of soluble components from the aligner matrix [25]. 

Leaching of soluble components into immersion media, as indicated by increased UV–Visible absorbance, suggests potential chemical instability of thermoplastic aligner materials under acidic and alkaline conditions. However, the present spectrophotometric approach does not permit identification or quantification of specific compounds such as residual monomers or additives. Therefore, the observed absorbance changes likely reflect the release of a mixture of low-molecular-weight substances rather than a defined chemical entity. Consequently, no direct conclusions regarding toxicological or biocompatibility risks can be drawn from the present findings, and further studies employing advanced analytical techniques such as high-performance liquid chromatography or mass spectrometry would be required to characterize specific leached compounds. This is supported by literature emphasizing the need to understand leaching behaviour as part of a comprehensive assessment of polymer performance [3, 7, 21]. 

### Clinical significance

Orthodontists should be aware that chemical exposures encountered intraorally including acidic foods and beverages can alter aligner material properties within a typical wear period. These changes may affect fit, force transmission, clarity, and aesthetic quality, potentially influencing treatment outcomes and patient satisfaction. Advising patients to minimize contact with strongly acidic or staining agents and encouraging adherence to prescribed cleaning protocols may mitigate undesirable material changes [26]. 

### Limitations and future scope

This study was conducted under controlled in vitro conditions, which do not fully replicate the complex and dynamic oral environment. Factors such as salivary flow, enzymatic activity, masticatory forces, temperature fluctuations, and microbial biofilms may influence aligner material behaviour in vivo and were not simulated in this model. Although three commercially available aligner materials were included, the study was designed to evaluate chemical exposure effects under standardized conditions rather than to perform an exhaustive inter-brand performance ranking. While a statistically powered sample size (*n* = 10 per group) was employed, results may not directly translate to clinical conditions. However, UV–Visible spectrophotometry is a well-established and validated preliminary screening method for detecting relative leaching trends of low–molecular-weight components from thermoplastic orthodontic materials under controlled in vitro conditions. These limitations should be considered when interpreting the findings, and future studies incorporating dynamic oral simulation and advanced analytical methods are recommended. Future research should incorporate clinical in-use evaluations, combining mechanical loading, salivary enzyme activity, and dietary exposures to better simulate real usage conditions. Longitudinal studies assessing how these physicochemical changes correlate with mechanical force delivery and treatment efficiency will further clarify their clinical implications. Comparative studies across different aligner materials and manufacturing processes (e.g., PETG vs. polyurethane or 3D-printed polymers) could inform material selection and design for improved performance and longevity [27]. Future investigations should employ advanced analytical techniques such as high-performance liquid chromatography–mass spectrometry (HPLC–MS) to identify and quantify specific compounds released from aligner materials.

## Conclusion

In vitro chemical exposure significantly impacts the physical and aesthetic properties of thermoplastic aligners over a 14-day period, with acidic environments causing the most pronounced changes in weight, colour stability, and leaching behaviour. These findings underscore the importance of understanding material responses to common oral exposures, guiding clinicians in patient education and material selection to optimize aligner performance and aesthetic outcomes.

## Data Availability

The datasets generated and analysed during the current study, including raw triplicate measurements for weight, pH, and absorbance, are available from the corresponding author upon reasonable request.

## Abbreviations

PETG: polyethylene terephthalate glycol
TPU: thermoplastic polyurethane
